# Microcirculation dysfunction in endotoxic shock rabbits is associated with impaired S-nitrosohemoglobin-mediated nitric oxide release from red blood cells: a preliminary study

**DOI:** 10.1186/s40635-018-0215-0

**Published:** 2019-01-07

**Authors:** Bo Yao, Da-Wei Liu, Wen-Zhao Chai, Xiao-Ting Wang, Hong-Min Zhang

**Affiliations:** 1 Department of Critical Care Medicine, Affiliated Hospital of Qingdao University, Qingdao, 266000 China; 2 Department of Critical Care Medicine, Peking Union Medical College Hospital, Peking Union Medical College, Chinese Academy of Medical Sciences, Beijing, 100730 China

**Keywords:** Sepsis shock, Red blood cell, S-nitrosohemoglobin, Microcirculation, Blood flow heterogeneity

## Abstract

**Background:**

Microcirculation dysfunction with blood flow heterogeneity is an important characteristic in sepsis shock. We hypothesized that impaired ability of red blood cells to release nitric oxide resulted in microcirculation dysfunction in sepsis shock.

**Methods:**

4,4′-Diisothiocyanatostilbene-2,2′-disulfonic acid disodium salt hydrate (DIDS), an inhibitor of band3 protein, was used to inhibit S-nitrosohemoglobin-mediated nitric oxide release. Rabbits were randomly divided into four groups: control (*n* = 6), lipopolysaccharide (LPS) (*n* = 6), LPS + DIDS (*n* = 6), and control + DIDS group (*n* = 6). Macrocirculation (cardiac output and mean arterial pressure) and microcirculation (microvascular flow index and flow heterogeneity index) parameters were recorded. At 2-h time point, arterial and venous S-nitrosohemoglobin concentrations were measured.

**Results:**

The arterial–venous difference for S-nitrosohemoglobin in the LPS group was lower than the control group (27.3 ± 5.0 nmmol/L vs. 40.9 ± 6.2 nmmol/L, *P* < 0.05) but was higher than the LPS + DIDS group, with a statistically significant difference (27.3 ± 5.0 nmmol/L vs. 16.0 ± 4.2 nmmol/L, *P* < 0.05). Microvascular flow index for the LPS group at 2 h was lower than the control group (1.13 ± 0.16 vs. 2.82 ± 0.08, *P* < 0.001) and higher than the LPS + DIDS group (1.13 ± 0.16 vs. 0.84 ± 0.14, *P* < 0.05). Flow heterogeneity index for the LPS group at 2 h was higher than the control group (1.03 ± 0.27 vs. 0.16 ± 0.06, *P* < 0.001) and lower than the LPS + DIDS group (1.03 ± 0.27 vs. 1.78 ± 0.46, *P* < 0.001).

**Conclusions:**

In endotoxic shock rabbits, the ability of S-nitrosohemoglobin-mediated nitric oxide release from RBC was impaired, and there was an association between the ability and microcirculation dysfunction especially increased blood flow heterogeneity.

**Electronic supplementary material:**

The online version of this article (10.1186/s40635-018-0215-0) contains supplementary material, which is available to authorized users.

## Background

Sepsis is common in intensive care units with high mortality [1]. Sepsis shock is the most severe manifestation of sepsis, with a mortality rate > 40% [1]. Microcirculation dysfunction with blood flow heterogeneity is an important characteristic in sepsis shock [2–4]. In sepsis shock, persistent microcirculatory dysfunction was reported to be associated with organ failure and mortality [5].

Treatments for sepsis shock in hemodynamics involve three aspects: macrocirculation, microcirculation, and cellular metabolism. In many cases, when macrocirculation indexes including cardiac output and blood pressure recover to normal values, tissue hypoxia persists [6]. This may be the reason for incoherence between the macrocirculation and microcirculation [7]. The goal of correcting macrocirculation through fluid resuscitation, vasoactive drug infusion, and cardiac inotrope infusion is to improve microcirculation. However, sometimes after septic shock resuscitation, no obvious improvements were observed in microvascular blood flow because of the increased flow heterogeneity with sepsis [8]. Other than macrocirculation, and although microcirculation and cell function can influence each other, it is reported that treatment aimed at the microcirculation may be more important in septic shock resuscitation [9]. Additionally, microcirculation dysfunction may occur earlier than mitochondria dysfunction in endotoxemia [10]. These findings support the importance of microcirculation dysfunction in sepsis shock [2, 3]. Therefore, correcting microcirculation dysfunction is a key aspect in sepsis shock resuscitation.

One of the mechanisms behind increased blood flow heterogeneity of microcirculation is maldistribution of nitric oxide (NO) caused by endothelial cell injury in sepsis [11]. Some researchers have targeted endothelial cells and inhibited NO synthase activity, but the results are controversial [12, 13]. Additionally, other studies have tried to open microvessel stenosis or occlusion to improve microcirculation through inhalation of NO, but without success [14]. The reason for improving the microcirculation is to get more red blood cells (RBC) into the hypoxic area, which provides more oxygen and alleviates tissue hypoxia. To achieve this, increased blood flow is needed, which requires dilation of occluded or narrowed vessels. It has been reported that RBCs themselves have the ability to dilate vessels independent of endothelium cells [15].There are three ways that RBC induce hypoxic vasodilation: the adenosine triphosphate (ATP) pathway, the nitrite pathway, and the S-nitrosohemoglobin (SNO-HB) pathway [16]. A characteristic of the SNO-HB pathway is that RBC can distribute NO active substances with the help of band3 protein according to demand and targets hypoxic vasodilation [16]. RBC can dilate the vessels with less flow, and increased numbers of RBC along with increased blood flow provides more oxygen to improve tissue hypoxia. Therefore, RBC may have an important role in the circulatory regulation, possibly.

We hypothesized that impairment of the SNO-HB pathway and the consequent RBC ability to release NO is a key factor behind microcirculation dysfunction in sepsis shock, in particular, flow heterogeneity. To this end, we studied the role of the SNO-HB pathway and the ability of RBC to release NO in the microcirculation in sepsis shock using endotoxin shock rabbits.

## Materials and methods

### Animals

Study protocols were approved by the Animal Care Committee of our hospital. Healthy specific-pathogen-free Japanese big-ear rabbits weighing 2.5–3.0 kg were housed individually at the animal resources unit at the Animal Research Institute of our hospital. Twenty-four Japanese big-ear rabbits were randomly divided into 4 groups: control group (*n* = 6), lipopolysaccharide (LPS) group (*n* = 6), LPS + 4,4′-diisothiocyanatostilbene-2,2′-disulfonic acid disodium salt hydrate (DIDS, a band3 protein inhibitor) group (*n* = 6), and control + DIDS group (*n* = 6).

### Surgical procedure

Infusion doses of 2% pentobarbital sodium (2.5 ml/kg) were administered through the outer vein on the edge of the ear for anesthesia. A left central intravenous catheter (4FR, 22GA (13 cm); Arrow, USA) was inserted 5 cm into the jugular vein. A left arterial catheter (20G; BD, USA) was inserted 2 cm into the internal carotid artery. After tracheotomy, rabbits were ventilated (Engström Carestation; Datex-Ohmeda (GE Healthcare), USA) with a fraction of inspired oxygen of 50%, a tidal volume of 10 ml/kg, a positive end-expiratory pressure of 0 cmH_2_O, and a respiratory rate of 35 breaths/min.

### Macrocirculation monitoring

The left jugular central intravenous catheter was used to monitor central venous pressure. The left jugular arterial catheter was used to monitor mean arterial pressure (MAP). An ultrasound (probe 6–13 HZ, M-Turbo® ultrasound system; Sonosite International, Washington, USA) was used to monitor cardiac output. The probe was located at the left side of the sternum with orientation for heart transection to obtain the left ventricular short axis plane. The left ventricular end-diastolic dimension and left ventricular end-systolic dimensions were recorded three times and means calculated.

The heart was considered to be a prolate ellipsoid with the left ventricular long axis (LA) dimension equal to twice the left ventricular short axis (SA) dimension. According to a prolate ellipsoid formula for measuring left ventricular volume, left ventricular volume = 4/3 * pi * SA squared * LA = 4/3 * pi * LA squared / 4 * LA ≈ LA cubed. So stroke volume = left ventricular end-diastolic volume − left ventricular end-systolic volume = left ventricular end-diastolic dimension cubed − left ventricular end-systolic dimension cubed. And cardiac output was obtained using the formula: cardiac output = stroke volume × heart rate.

### Microcirculation monitoring

A 3-cm patch of fur close to the tip of the ear was removed and 2–3 drops of pine tar were applied to the area. A microcirculation monitor (TR8000B; Peking Tongrentaikang Medical Equipment Co., China) was fixed to the tip of the ear, which allowed for observation of ear microcirculation by adjusting parameters (Additional file 1). Close to 20 s of microcirculation video was recorded using the software kit. Following guidelines [17], the obtained image was divided into four quadrants and for each quadrant the predominant flow (absent = 0, intermittent = 1, sluggish = 2, and normal = 3) was assessed. The microvascular flow index (MFI) score equals the averaged values of the four quadrants. For five random images, the flow heterogeneity index (FHI) was determined by dividing (MFI max − MFI min) by MFI mean.

### Experimental protocol

At 15 min after surgery, rabbits in the LPS + DIDS and control + DIDS group were intravenously infused with band3 protein inhibitor (DIDS, Product No. D3514; Sigma-Aldrich, St. Louis, MI, USA) dissolved in 0.1 mmol/L KHCO_3_ at a dose of 60 mg/kg. Rabbits in the other groups were intravenously administered an equal volume of 0.1 mmol/L KHCO_3_. Rabbits in the LPS and LPS + DIDS groups were then intravenously administered LPS from *Escherichia coli* 055:B5 (Product No. L2880; Sigma-Aldrich) at a dose of 2 mg/kg for 1 min. Rabbits in the control and control + DIDS group were intravenously administered an equal volume of 0.9% saline for 1 min.

Hemodynamic parameters (mean arterial pressure, cardiac output, MFI, and FHI) for each group were recorded. At 2-h intervals, a series of tests were conducted, which included arterial and venous SNO-HB, band3 protein content in RBC. Band3 amount was measured by ELISA kit (Product No. QY-Q8642; Qiaoyu-shanghai, china) following the manufacturer’s instructions. The concentration of SNO-HB was measured by photolysis chemiluminescence methods [18]. Following tests, rabbits were killed by air embolism and death was confirmed by monitoring the ECG for 30 min.

### The measurement of SNO-HB by photolysis chemiluminescence method

The blood sample was collected in anticoagulation tube and centrifuged (500 r/min, 2 min) to obtain RBCs with removing the supernatant. RBCs were washed in phosphate-buffered saline (PBS) at pH 7.4 and centrifuged twice again. Then RBCs were lysed in 0.1 mmol/L ethylene diamine tetraacetic acid (EDTA) (1:10 volume ratio, 10 min). Lysates were centrifuged (2000 r/min for 10 min) and filtered. Then lystates were purified by gel filtration over a Sephadex G-25 spin columns (GE Healthcare) equilibrated with PBS (pH 7.40, with 0.5 mmol/L EDTA). Hemoglobin samples were then stored at − 80 °C for batch analysis. The sample was incubated with either PBS or sixfold molar excess of mercuric chloride. Then the sample mixed with a helium carrier stream was infused into a borosilicate glass coil by a HPLC capillary pump. The coil was illuminated with a UV light (200 W mercury vapor lamp) for photolysis of NO. And then they were carried into a chemiluminescence analyzer (Thermo Electron Corp. TEA 610). Concentration of SNO-HB was calculated by the difference between the amount of NO in the absence and presence of mercuric chloride [18].

### Statistical analysis

The statistical analysis was performed using SPSS 17.0 software (SPSS, Inc., Chicago, IL, USA). In quantitative data, the results were expressed as mean ± standard deviation. We used one-way analysis of variance to test differences among three groups and Bonferroni (B) methods to compare each other. A value of *P* < 0.05 was considered statistically significant.

## Results

After infusion of LPS, blood pressure began to drop at 30 min. Blood pressure dropped to below 65 mmHg at 2 h in all animals, which suggested that the animal model of endotoxin shock was established.

There was no difference in cardiac output between the four groups (0.186 ± 0.046 L/min vs. 0.201 ± 0.030 L/min vs. 0.169 ± 0.033 L/min vs. 0.193 ± 0.021 L/min, *P* > 0.05). The value of MAP in LPS group was lower than the control group at 2 h (57.7 ± 6.5 mmHg vs. 69.0 ± 4.5 mmHg, *P* < 0.05). MFI for the LPS group at 2 h was lower than the control group (1.13 ± 0.16 vs. 2.82 ± 0.08, *P* < 0.001) and higher than the LPS + DIDS group (1.13 ± 0.16 vs. 0.84 ± 0.14, *P* < 0.05). MFI for the control + DIDS group at 2 h was lower than the control group (2.44 ± 0.23 vs. 2.82 ± 0.08, *P* < 0.001). FHI for the LPS group at 2 h was higher than the control group (1.03 ± 0.27 vs. 0.16 ± 0.06, *P* < 0.001) and lower than the LPS + DIDS group (1.03 ± 0.27 vs. 1.78 ± 0.46, *P* < 0.01). FHI for the control + DIDS group at 2 h was higher than the control group (0.60 ± 0.17 vs. 0.16 ± 0.06, *P* < 0.01) (Table 1).

**Table 1:** The hemodynamic indexes among four groups

Indexes	Control group	LPS group	LPS + DIDS group	Control + DIDS group
MAP (mmHg)	69.0 ± 4.5^#^	57.7 ± 6.5	46.7 ± 9.3	69.0 ± 6.3
CO (L/min)	0.186 ± 0.046	0.201 ± 0.030	0.169 ± 0.033	0.193 ± 0.021
MFI	2.82 ± 0.08^#^	1.13 ± 0.16	0.84 ± 0.14^#^	2.44 ± 0.23^#^*
FHI	0.16 ± 0.06^#^	1.03 ± 0.27	1.78 ± 0.46^#^	0.60 ± 0.17*

The sign # represented the value in the group had statistical difference compared with LPS group (*P*<0.05); the sign* represented the value in the control + DIDS group had statistical difference compared with control group (*P*<0.05). *MAP* mean arterial pressure, *CO* cardiac output, *MFI* microvascular flow index, *FHI* flow heterogeneity index

**Fig. 1:**
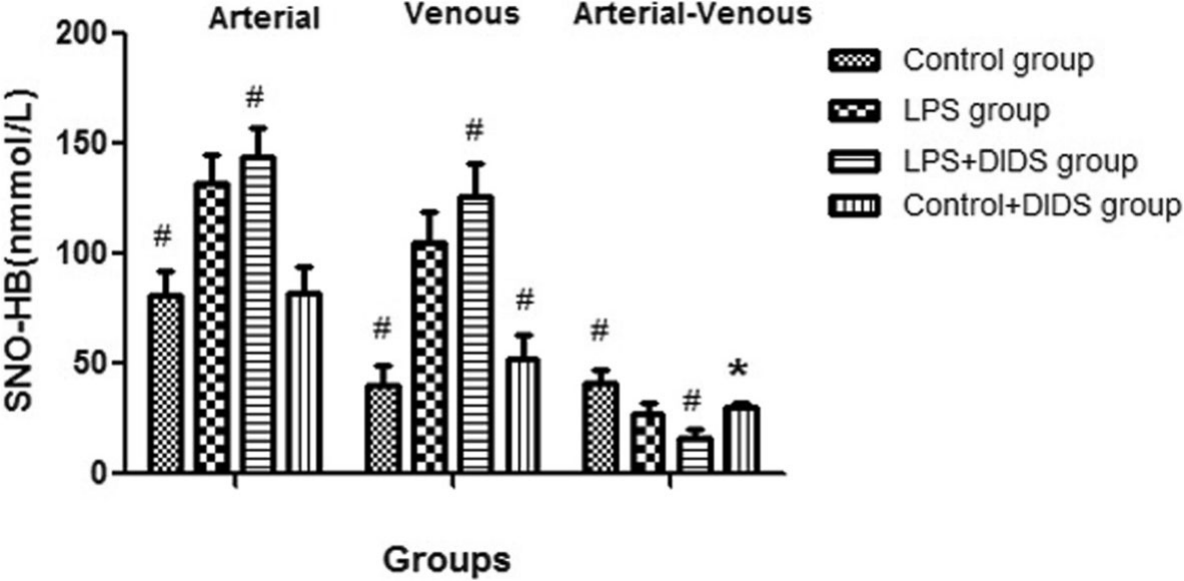
The arterial and venous SNO-HB concentrations among four groups. The sign # represented the value in the group had statistical difference compared with LPS group (*P* < 0.05); the sign * represented the value in the control + DIDS group had statistical difference compared with control group (*P* < 0.05)

The arterial–venous difference for SNO-HB in the LPS group was lower than the control group (27.3 ± 5.0 nmmol/L vs. 40.9 ± 6.2 nmmol/L, *P* < 0.05) but was higher than the LPS + DIDS group, with a statistically significant difference (27.3 ± 5.0 nmmol/L vs. 16.0 ± 4.2 nmmol/L, *P* < 0.05). The arterial–venous difference for SNO-HB in the control + DIDS group was also lower than the control group (30.5 ± 2.1 nmmol/L vs. 40.9 ± 6.2 nmmol/L, *P* < 0.05) (Fig. 1).

There was no significant difference in band3 protein between four groups (350.8 ± 37.4 μg/ml vs. 345.8 ± 24.5 μg/ml vs. 349.7 ± 17.9 μg/ml vs. 345.3 ± 22.6 μg/ml, *P* > 0.05).

## Discussion

In this study, we found that the ability of RBC to release NO via the SNO-HB pathway was impaired in endotoxic shock rabbits. The microcirculation dysfunction became worse, especially heterogeneity of microcirculation showed a marked increase, after SNO-HB-mediated NO release was inhibited by DIDS with no change of macrocirculation.

RBC may promote vasodilatation via the SNO-HB pathway. When RBC arrives at the lung, not only oxygen but also NO can bind to hemoglobin. Following transnitrosation, hemoglobin is transformed into SNO-HB. When RBC reaches hypoxic tissue, hemoglobin undergoes a conformational transition, and oxygen detaches from hemoglobin. Additionally, the free NO in SNO-HB moves out though the RBC membrane via band3 protein and is turned into RSNO in the plasma. The role of RSNO in vasodilatation is to increase the number of RBC at the site. These processes suggest that SNO-HB-mediated NO release from RBC is vasodilatation-targeted [16].

In the current study, the arterial–venous gradient for SNO-HB was used to determine SNO-HB-mediated NO release from RBC [19, 20]; MFI and FHI was used to determine the microcirculation [17]. We found that the arterial–venous gradient gap for SNO-HB was less in endotoxic shock, suggesting impairment in the process. Microcirculation dysfunction also occurs in endotoxic shock, so it is possible that there is an association between microcirculation dysfunction and SNO-HB-mediated NO release from RBC. However, there are also other factors that influence microcirculation in septic shock, blood pressure, for example. After band3 protein was inhibited by DIDS, SNO-HB-mediated NO release from RBC decreased, and without alterations of the other macrocirculation parameters (MAP and CO), microcirculation was still worse. This further suggests the impairment of SNO-HB-mediated NO release from RBC results in microcirculation dysfunction in endotoxic shock.

The release of NO active substances according to requirements and targeted dilation of vessel are characteristic of SNO-HB-mediated NO release from RBC [16]. In sepsis shock, maldistribution of NO can result in microcirculation dysfunction with flow heterogeneity [11]. It is possible that RBC can redistribute NO according to requirements and can target the relaxation of hypoxic vessels. This allows for increased numbers of RBC to reach hypoxic tissue through increased blood flow, and, consequently, more oxygen can be released from the increased numbers of RBC to improve tissue hypoxia. The results of the current study suggest that this ability of RBC was impaired in our animal model of sepsis shock, and RBC may not be able to remedy or directly result in microcirculation dysfunction, and could be a reason for the association between microcirculation dysfunction and SNO-HB-mediated release of NO from RBC.

Actually, some previous researches about SNO-HB were highly controversial [21–25]. Isbell et al. found that the blood pressure and time-to-fatigue in exercise were not significantly changed in the βCys93 mutant and wild-type mice, so they concluded that SNO-HB was not essential for hypoxic vasodilation [21]. Soon after, this conclusion was disputed because systemic blood pressure and time-to-fatigue in exercise were not direct parameters about hypoxic vasodilation [22]. In another research, Zhang et al. monitored both local blood flow and tissue oxygenations in a knockin mouse model and clearly demonstrated that SNO-HB played an essential role in hypoxic vasodilation [23]. Bailey DM et al. agreed that SNO-HB had no role in vasodilation based on V > A SNO-HB gradient [24]. But Stamler JS doubted that the detail of SNO-HB monitoring was ignored in this study [25]. SNO-HB gradients may be reversed when venous and “hypoxic” samples were exposed to room air because the disposition of NO within hemoglobin was regulated by oxygen partial pressure [26]. There are few studies examining the SNO-HB pathway in sepsis. Morgan MA et al. compared concentrations of arterial–venous gradient for SNO-HB and nitrite in 87 sepsis patients and 52 healthy volunteers and found that the gap of the arterial–venous gradient for SNO-HB reduced [20]; a finding similar to our own results.

Band3 protein is an anion-exchanger and important membrane protein in RBC. When the bio-activator NO is released from RBC via the SNO-HB pathway, band3 protein is required as a transporter [27]. In the current study, total amounts of band3 protein showed no significant differences between control and LPS group; a finding similar to a previous study [28]. Total amounts of band3 protein also remained stable after administration of DIDS (a band3 protein inhibitor). However, microcirculation assuredly worsened. We suggest that it is possible that the function of brand3 protein may be impaired. In a mouse model of sepsis, band3 protein was found to be phosphorylated [29], and a cluster of band 3 erythrocytes were found in exhaustive exercise mice [30]. It is therefore possible that while the amount of band3 protein may remain stable, whether the structure of band3 protein clusters in erythrocytes or its function change (via phosphorylation, for example) requires further study.

This study had some limitations. First, band3 protein is essentially an anion channels exchanger protein. DIDS not only inhibits NO transport function but also the anion exchanger function. Anion exchanger can regulate intracellular pH, which is an important regulator of vascular smooth muscle cell tone. Actually, mRNA and proteins of anion exchanger 2 and 3 expressed in vascular smooth muscle cells and microvessels [31]. So DIDS was a non-specific inhibitor and we cannot eliminate the effect of anion exchange function for the results. Moreover, other than band3, protein disulfide isomerase can directly participate in the efflux of NO from erythrocytes [32]. In this study, we only intervened band3 protein, so the effect of SNO-HB may be partly inhibited. Second, we provided fluids to keep CVP in a normal value. But we actually provided no vasoactive agents to keep the normal blood pressure. So blood pressure may be a confounder. Third, we used the concentration of arterial–venous SNO-HB to evaluate the release of NO from RBC via the SNO-HB pathway assuming that SNO-HB is eliminated continuously from arterial to venous blood similar to oxygen. However, it was also not an accurate parameter to reflect the ability of SNO-HB. Generally, advanced studies are needed to confirm the results.

## Conclusions

In this rabbit model of endotoxic shock, the ability of SNO-HB-mediated NO release from RBC was impaired, and there is an association between the impaired ability and microcirculation dysfunction especially increased flow heterogeneity of microcirculation in endotoxic shock. It needed further study to confirm that the SNO-HB pathway is a key factor of sepsis microcirculation dysfunction.

## Additional file

Additional file 1: Microcirculation of rabbit ear × 200. (AVI 7741 kb)

## Abbreviations

DIDS: 4,4′-Diisothiocyanatostilbene-2,2′-disulfonic acid disodium salt hydrate
SNO-HB: S-nitrosohemoglobin
RBC: Red blood cells
NO: Nitric oxide
MFI: Microvascular flow index
FHI: Flow heterogeneity index
ATP: Adenosine triphosphate
LPS: Lipopolysaccharide
RSNO: S-nitrosothiols
MAP: Mean arterial pressure
